# Dihydroisotanshinone I and BMAL-SIRT1 Pathway in an In Vitro 6-OHDA-Induced Model of Parkinson’s Disease

**DOI:** 10.3390/ijms241311088

**Published:** 2023-07-04

**Authors:** Hui-Chen Su, Yuan-Ting Sun, Ming-Yu Yang, Ching-Yuan Wu, Cheng-Ming Hsu

**Affiliations:** 1 Department of Neurology, National Cheng Kung University Hospital, College of Medicine, National Cheng Kung University, Tainan 70101, Taiwan; n510807@mail.hosp.ncku.edu.tw (H.-C.S.); ytsun@mail.ncku.edu.tw (Y.-T.S.); 2 Graduate Institute of Clinical Medical Sciences, College of Medicine, Chang Gung University, Taoyuan 33302, Taiwan; yangmy@mail.cgu.edu.tw; 3 Department of Otolaryngology, Kaohsiung Chang Gung Memorial Hospital, Kaohsiung 83301, Taiwan; 4 Department of Chinese Medicine, Chiayi Chang Gung Memorial Hospital, Chiayi 61363, Taiwan; 5 Department of Otolaryngology-Head and Neck Surgery, Chiayi Chang Gung Memorial Hospital, Chiayi 61363, Taiwan; 6 School of Medicine, College of Medicine, Chang Gung University, Taoyuan 33302, Taiwan; 7 Cancer Center, Chiayi Chang Gung Memorial Hospital, Chiayi 61363, Taiwan

**Keywords:** Idiopathic Parkinson’s disease, Danshen, Dihydroisotanshinone I, 6-OHDA, *SIRT1*, *BMAL1*, reactive oxygen species, ROS

## Abstract

Danshen has been widely used for the treatment of central nervous system diseases. We investigated the effect of dihydroisotanshinone I (DT), a compound extracted from Danshen, as well as the corresponding mechanisms in an in vitro-based 6-OHDA-induced Parkinson’s disease (PD) model. SH-SY5Y human neuroblastoma cell lines were pretreated with 6-hydroxydopamine (6-OHDA) and challenged with DT. Subsequently, the cell viability and levels of reactive oxygen species (ROS) and caspase-3 were analyzed. The effect of DT on the 6-OHDA-treated SH-SY5Y cells and the expression of the core circadian clock genes were measured using a real-time quantitative polymerase chain reaction. Our results indicated that DT attenuated the 6-OHDA-induced cell death in the SH-SY5Y cells and suppressed ROS and caspase-3. Moreover, DT reversed both the RNA and protein levels of *BMAL1* and *SIRT1* in the 6-OHDA-treated SH-SY5Y cells. Additionally, the *SIRT1* inhibitor attenuated the effect of DT on *BMAL1* and reduced the cell viability. The DT and *SIRT1* activators activated *SIRT1* and *BMAL1*, and then reduced the death of the SH-SY5Y cells damaged by 6-OHDA. SIRT1 silencing was enhanced by DT and resulted in a BMAL1 downregulation and a reduction in cell viability. In conclusion, our investigation suggested that DT reduces cell apoptosis, including an antioxidative effect due to a reduction in ROS, and regulates the circadian genes by enhancing SIRT1 and suppressing BMAL1. DT may possess novel therapeutic potential for PD in the future, but further in vivo studies are still needed.

## 1. Introduction

Idiopathic Parkinson’s disease (iPD) is a complex neurodegenerative disorder caused by the progressive decline of dopaminergic neurons in the midbrain [1]. The therapeutic strategies for iPD, including medication, physiotherapy, music therapy, and deep brain stimulation, mainly focus on maintaining neuronal function. However, due to its multifactorial pathophysiology, there are currently seldom proven neuroprotective therapies for iPD [2]. The goals of the pharmacologic treatments for iPD are to restore the dopaminergic activity in the striatum and maintain the balance between the dopaminergic and cholinergic effects to improve the associated motor deficits and avoid long-term motor and non-motor complications [2,3]. The medications for iPD include dopamine precursors (levodopa and carbidopa/levodopa combination), non-ergot dopamine agonists (pramipexole, ropinirole, and rotigotine), a catechol-O-methyltransferase inhibitor (entacapone), and monoamine oxidase-B (MAO-B) inhibitors (selegiline and rasagiline) [4]. However, these medications lack long-term effectiveness due to the progressive neurodegenerative process of the disease. Therefore, novel alternatives are needed to address the inadequate efficacy of conventional antiparkinsonian drugs.

Patients who are newly diagnosed with Parkinson’s disease (PD) often report sleep problems that reduce their quality of life [5]. The sleep cycle and circadian rhythms are mainly controlled by the suprachiasmatic nucleus of the anterior hypothalamus. Motor activities and environmental cues, such as light and food intake, tune the circadian rhythm to a 24-h cycle [5]. The circadian rhythm system is present in nearly all organisms and coordinates behavioral and physiological reactions to changes in the surrounding environment [6]. At the molecular level, the circadian rhythm system is mainly composed of a group of conserved clock proteins, including the brain and muscle Arnt-like protein 1 (*BMAL1*), *CLOCK*, periodic circadian rhythm proteins (*PER1–3*), and cryptochromes (*CRY1* and *CRY2*) [7]. Dopamine modulates the *BMAL1/CLOCK* heterodimer activity, and its deficiency may directly affect this central component of the molecular clock, resulting in the dysfunction of the *BMAL1* expression in PD [8]. *SIRT1*uins, silent information regulators (SIRs), are a family of nicotine adenine dinucleotide (NAD+)-dependent protein deacetylases or adenosine diphosphate-ribosyltransferases. Studies have shown that *SIRT1* links the cellular metabolism to the circadian clock genes [9,10].

Due to its neurotoxicity, 6-Hydroxydopamine (6-OHDA) is commonly used as an inducer of neurodegeneration in in vitro models of PD [11]. The structural analog of dopamine, 6-OHDA, selectively uptakes noradrenergic and dopaminergic neurons by the norepinephrine transporters and membranous dopamine, respectively. In neurons, 6-OHDA accumulates in the cytosol, where it is metabolized into dihydrophenylacetic acid or oxidized into hydrogen peroxide and p-quinone with the participation of MAO enzymes. This process leads to the formation of reactive oxygen species (ROS) and oxidative stress in neurons, then causes cell death [12,13,14].

Danshen (*Salvia miltiorrhiza*) is a common Chinese herbal medicine that has been widely used for over 1000 years [15]. The main components of Danshen are hydrophilic phenolic acids and lipophilic compounds [16] that contribute to its antioxidant and anti-inflammatory properties, as well as its therapeutic properties, such as its usage in cardiovascular disease treatment [17]. Tanshinone, a lipophilic compound of Danshen, has been classified into four types based on its structural diversity: tanshinone IIA, cryptotanshinone, tanshinone I (Tan I), and dihydroisotanshinone I (DT) [18]. In clinical practice, Danshen is used for the treatment of various diseases, including cardiovascular diseases [19], cerebrovascular diseases [18,20], Alzheimer’s disease [21], PD [22], and renal injury [23]. Danshen is widely used for the treatment of cardiovascular disorders and its aqueous extract has shown anticancer as well as antioxidant effects [19]. Moreover, Danshen has been widely used in the treatment of central nervous system diseases. For instance, a study demonstrated that Tan I inhibits the expression of proinflammatory genes in activated microglia [24]. Neuroprotective agents as part of PD treatment may help delay or slow the disease progression [25].

In this study, we aimed to verify the effect of DT and investigate its underlying mechanisms using an in vitro-based 6-OHDA-induced PD model.

## 2. Results

### 2.1. Effect of DT on the 6-OHDA-Induced Cytotoxicity

**Figure 1:**
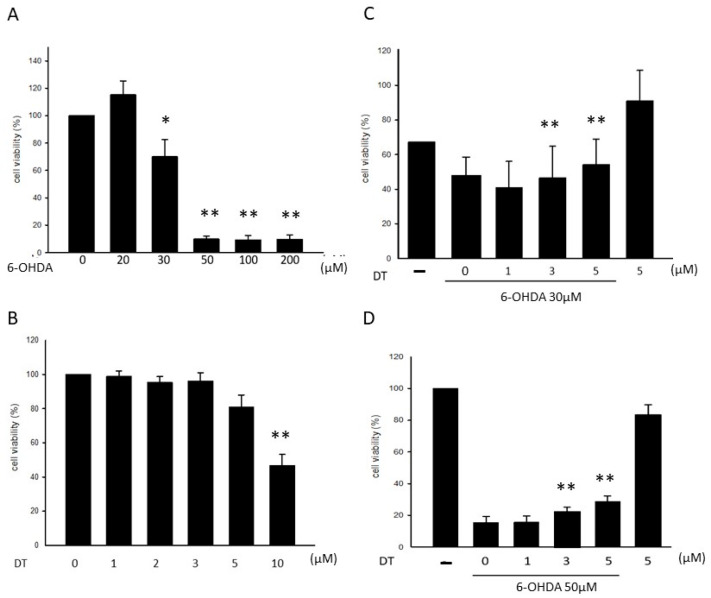
Effects of the different concentrations of 6-OHDA and DT on the SH-SY5Y cell viability. The treatment of the cells using 6-OHDA (**A**) (30 μM or 50 μM) or DT (5 μM) (**B**) for 24 h and the subsequent measurement of the cell viability using the MTT assay. The results showed that 6-OHDA reduced the cell viability, whereas 5 μM of DT exhibited a minimal toxic effect. The protective effects of DT against 30 (**C**) and 50 (**D**) μM of 6-OHDA induced SH-SY5Y cell damage. * *p* < 0.05, ** *p* < 0.01, indicate the level of significant difference between the DT-treated and DT-untreated cells prior to the 6-OHDA treatment.

For the induction of the neurotoxicity, we treated the cells with different concentrations of 6-OHDA for 24 h and 48 h, and we analyzed the cell viability using the 3-(4,5-dimethylthiazol-2-yl)-2,5-diphenyl-2H-tetrazolium bromide (MTT) assay. We observed that 6-OHDA significantly decreased the cell viability starting at a concentration of 20 μM, followed by 30, 50, 100, and 200 μM, resulting in the lowest cell viability (Figure 1A). Based on this result, we used 30 and 50 μM of 6-OHDA to induce cytotoxicity in the subsequent experiments. We then tested the different concentrations of DT to confirm its toxicity. The treatment using 5 μM of DT did not significantly reduce the cell viability in the treated SH-SY5Y cells after 24 h (Figure 1B). For the succeeding experiment, we pretreated the SH-SY5Y cells with varying concentrations of DT for 12 h and induced them using 30 or 50 μM of 6-OHDA for 24 h. We observed that DT at the concentrations of 3 and 5 μM significantly attenuated the cell toxicity induced by 30 or 50 μM of 6-OHDA (30 μM, *p* = 0.0050, *p* = 0.0047; 50 μM *p* = 0.0003, *p* = 0.0007, respectively; Figure 1C,D).

### 2.2. Effect of DT on Cell Apoptosis and the 6-OHDA-Induced ROS Formation

**Figure 2:**
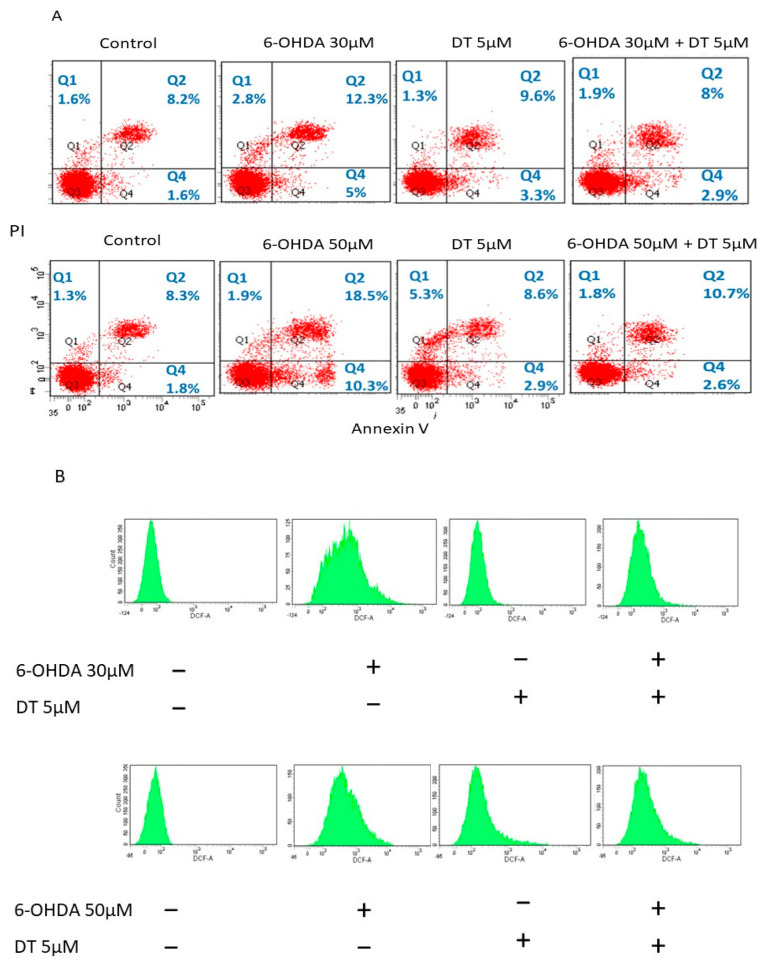

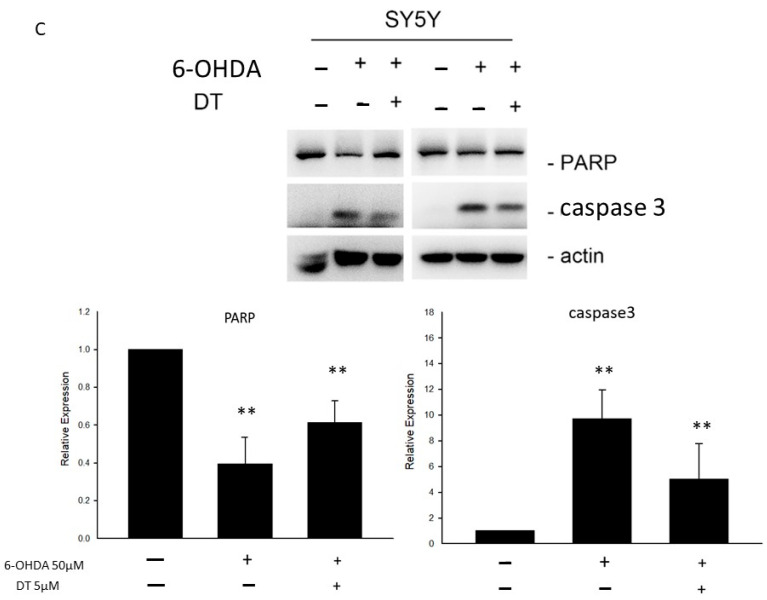
(**A**) bThe SH-SY5Y cells were kept as the control or treated with 6-OHDA (30 and 50 μM) for 24 h and the other two groups were only treated with DT 5 μM or a combination of 6-OHDA (30 or 50 μM) and DT 5 μM. Then, the cell apoptosis was examined using PI/Annexin V-FITC double staining and flow cytometry analysis. (**B**) The ROS formation in the 6-OHDA (30 μM or 50 μM)-treated SH-SY5Y cells and the ROS suppression in the 5 μM DT-treated SH-SY5Y cells. (**C**) The suppression of caspase-3 by DT in the 30 μM 6-OHDA-treated cells. ** *p* < 0.01 indicate the level of significant difference between the DT-treated and DT-untreated cells prior to the 6-OHDA treatment.

The SH-SY5Y cells were kept as the control, treated with 6-OHDA (30 or 50 μM), DT 5 μM, and 6-OHDA (30 or 50 μM) plus DT 5 μM for 24 h. Then, the cell apoptosis was examined using PI and Annexin V-FITC double staining and flow cytometry analysis. The development of the cell apoptosis was significantly manifested in both cells treated with 6-OHDA, which initiated a decrease in living cells and an increase in apoptotic and necrotic cells compared to the untreated controls (6-OHDA 30 μM:6-OHDA30 μM + DT 5 μM:control = 15.1%:8%:9.8%; 6-OHDA 50 μM:6-OHDA50 μM + DT 5 μM:control = 20.4%:12.5%:9.6 %; all *p* < 0.05). Quadrants 1 (Q1) and Quadrants 2 (Q2) were indicated as the late apoptotic and necrotic cells, Quadrants 3 (Q3) were indicated as the living cells, and Quadrants 4 (Q4) were indicated as the apoptotic cells (Figure 2A).

Compared to the untreated cells, those treated with 30 or 50 μM of 6-OHDA for 6 h had significantly higher levels of intracellular ROS (113.0% ± 36.76% vs. 2263.67% ± 196.5%, *p* = 0.0024). However, the pretreatment with DT 5 μM markedly decreased the levels of 6-OHDA-induced ROS (Figure 2B) and caspase-3 (Figure 2C).

### 2.3. Effect of DT on the PER1, CLOCK, BMAL1, and SIRT1 Levels in the 6-OHDA-Treated SH-SY5Y Cells

**Figure 3:**
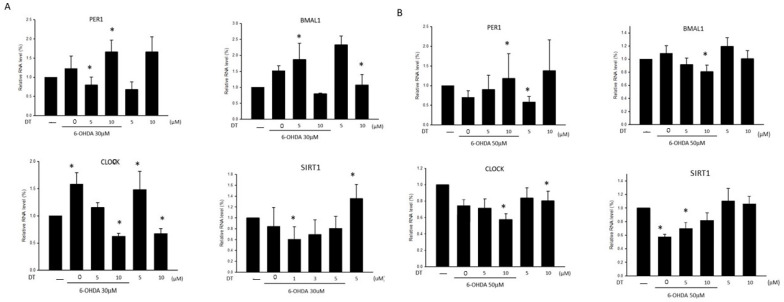
The *PER1*, *BMAL1*, *CLOCK*, and *SIRT1* expressions in the 30 (**A**) and 50 (**B**) μM 6-OHDA-treated SH-SY5Y cells before and after the 0, 5, and 10 μM DT treatments. The relative RNA level indicates the Log2-fold change of the mRNA levels relative to the control samples. * *p* < 0.05 indicate the RNA level of a significant difference between the DT treated with or without 6-OHDA and the control samples.

To investigate the mechanism of the 6-OHDA-induced disruption of the circadian clock genes, we measured the RNA levels of *PER1*, *CLOCK*, *BMAL1*, and *SIRT1* after the 6-OHDA treatment. Both the 30 μM or 50 μM 6-OHDA-treated SH-SY5Y cells exhibited reductions in the levels of *SIRT1* RNA and an elevation in the levels of *BMAL1* RNA. The pretreatment using 5 μM of DT for 12 h reversed the reduction in *BMAL1* and *SIRT1* RNA in the 30 μM 6-OHDA-treated cells (Figure 3A), whereas the pretreatment using 10 μM of DT for 12 h reversed the reduction in *PER1* and *SIRT1* RNA and reversed the elevation in *CLOCK* and *BMAL1* RNA in the 50 μM 6-OHDA-treated cells (Figure 3B). The *p*-values (Figure 3A,B) for *PER1*, *CLOCK*, *BMAL1*, and *SIRT1* were not all significant, but there was a trend toward a true statistical significance.

### 2.4. Effect of DT and the SIRT1 Inhibitor on the 6-OHDA-Induced Cell Toxicity

**Figure 4:**
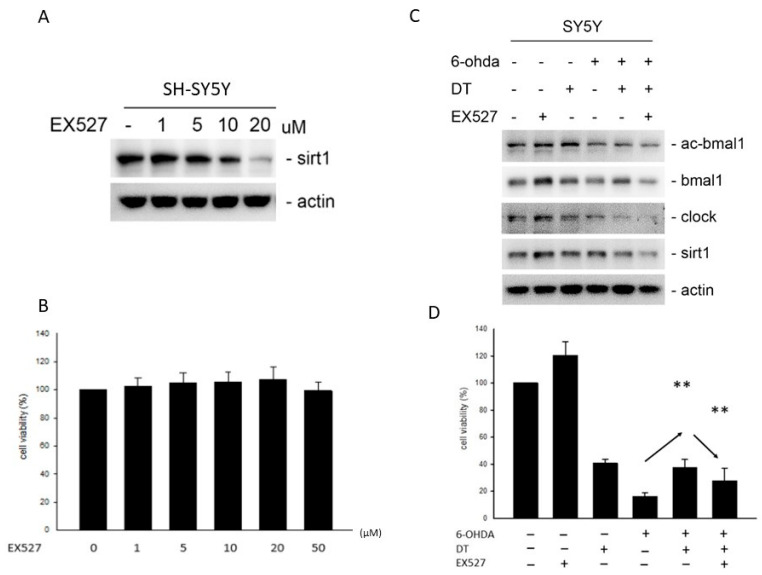
(**A**) EX-527 (*SIRT1* inhibitor) reduced the *SIRT1* expression. (**B**) The nil neurotoxic effect of EX-527 (1, 5, 10, 20 μM) (**C**) 5 μM of DT reversed the 6-OHDA-induced reduction in the *BMAL1* and *SIRT1* expression. EX-527 reversed the effect of DT on the *BMAL1* and *SIRT1* expression. (**D**) The DT treatment significantly reversed the 30 μM 6-OHDA-induced cell death (*p* = 0.0089). The reduction in the effect of 10 μM of DT using EX-527. ** *p* < 0.01 indicate the level of significant difference between the DT-treated and DT-untreated cells or the EX-527-treated and EX-527-untreated cells.

EX-527, an inhibitor of the deacetylase protein *Sirtuin* 1 (*SIRT1*), reduced the expression and activity of *SIRT1,* whose dysregulation resulted in neuronal damage in PD (Figure 4A). However, EX-527 did not exhibit neurotoxicity in the SH-SY5Y cells, as shown in Figure 4B. The pretreatment using 5 μM of DT for 12 h reversed the reduction in *SIRT1* and reversed the *BMAL1* proteins in the 6-OHDA-treated cells. However, the EX-527 treatment reversed the effect of DT on the *SIRT1* and *BMAL1* protein levels in the 6-OHDA-treated cells (Figure 4C). The treatment using 5 µM of DT significantly reversed the 6-OHDA-induced cell death (*p* = 0.0089), but the addition of 10 µThM of EX-527 weakened the cell viability (Figure 4D).

### 2.5. Effect of the DT, ROS, and SIRT1 Activators on BMAL1, SIRT1, and PARP after the 6-OHDA Treatment

**Figure 5:**
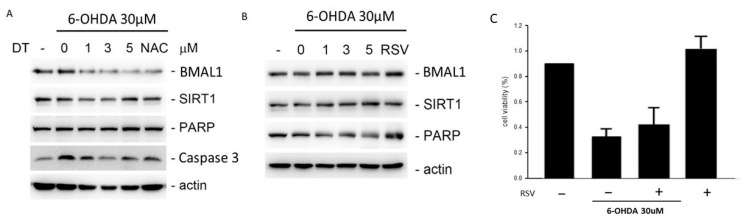
(**A**) DT as NAC, reduced the *BMAL1* and PARP expressions after the 30 µM 6-OHDA treatment. (**B**) DT, as RSV, increase the *BMAL1* and PARP expressions. (**C**) RSV increased the cell viability after the 6-OHDA treatment. (NAC: acetylcysteine; RSV: *SIRT1* activator).

To investigate whether the effect of DT was due to the ROS and *SIRT1* activation, we measured the protein levels of *BMAL1*, *SIRT1,* caspase 3, and *PARP* after the 6-OHDA treatment through pretreatment using 1, 3, and 5 μM of DT, acetylcysteine (NAC), or resveratrol (RSV, *SIRT1* activator) for 12 h. Reducing the ROS concomitantly inhibited the expression of *BMAL1, Caspase 3*, *PARP* but increased that of *SIRT1*. DT increased *SIRT1* and reduced the other proteins expression as NAC (Figure 5A). RSV, the *SIRT1* activator, increased the expression of *BMAL1, PARP*. (Figure 5B).

### 2.6. Effect of DT and SIRT1 siRNA on the BMAL1 Expression and Cell Viablity

**Figure 6:**
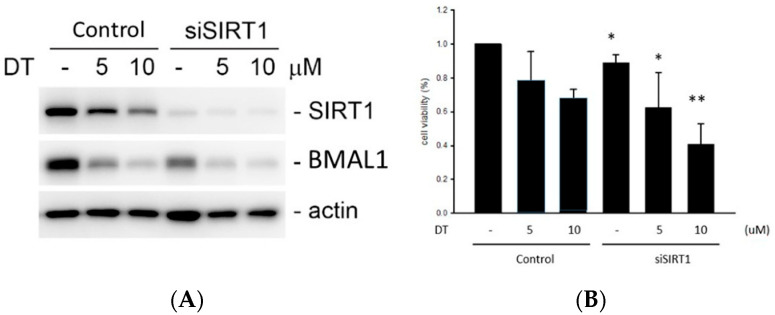
(**A**) siSIRT1 (SIRT1 small interfering RNA) silenced the SIRT expression. BMAL was also downregulated by siSIRT1. DT reduced SIRT1 and BMAL1. (**B**) SIRT1 silencing enhanced the reduction in the cell viability by DT. * *p* < 0.05 and ** *p* < 0.01 indicate the level of significant difference between the siSIRT1-treated and siSIRT1-untreated cells.

SIRT silencing was enhanced by DT and resulted in a BMAL1 downregulation (Figure 6A). SIRT1 silencing enhanced the reduction in the cell viability by DT. (Figure 6B).

## 3. Discussion

Idiopathic Parkinson’s disease results in major disability and high medical costs due to motor deficits and concurrent multisystem complications occurring over the course of the disease. Patients may not be aware of the disease until clinical motor symptoms occur. According to Braak’s theory, the pathological accumulation of Lewy bodies or similar compounds begins in the brainstem in patients with stage I or II PD, followed by its spread to the motor system in patients with stage III or IV PD and then to the neocortex in patients with stage V or VI PD, who often present dementia.

Currently, the effectiveness of therapeutic interventions to arrest PD and its progression remains unsatisfactory [26]. Since the introduction of levodopa for iPD treatment in the 1960s, pharmacological therapy has mainly focused on motor symptom management and neurological function restoration. Scientists have strived to identify the crucial pathways or indicators that would allow for early diagnosis and delay iPD progression. Neuroprotective agents, such as MAO-B inhibitors, dopamine receptor agonists, N-methyl-D-aspartate receptor antagonists, iron chelators, and other neurotrophic factors, are being investigated in experimental or clinical studies. However, varied results have been obtained [27].

Traditional antiparkinsonian drugs alleviate motor symptoms in early-stage patients. However, they are accompanied by several side effects and late-stage complications that can be debilitating to older patients and reduce their quality of life. The commonly reported side effects in older patients taking levodopa or dopamine receptor agonists include nausea, vomiting, confusion, postural hypotension, hallucinations, delusions, psychosis, and agitation [28]. As far as we know, several in vitro studies, in vivo studies, and clinical trials suggest that rasagiline, one of the MAO-B inhibitors, might exert neuroprotective effects by mitigating oxidative stress, increasing the expression of a glial cell line-derived neurotrophic factor [29], blocking the apoptosis pathway, and promoting the survival of dopaminergic neurons [30].

The complete pathogenesis of iPD remains unclear. However, studies have showed that multiple factors may be involved, including environmental factors, mitochondrial dysfunction, neuroinflammation, oxidative stress, and genetic factors [30,31,32,33,34]. Neuroinflammation is highly emphasized in neurodegenerative diseases, such as PD and Alzheimer’s disease, and is related to neurogenesis dysregulation [32,33]. Normal biological rhythms are essential for regulating antioxidant function. However, circadian rhythm disruptions are frequently noted in patients with iPD. These disruptions may result in the oscillation and fluctuation of motor and non-motor symptoms in iPD.

Oxidative stress is a crucial pathological mechanism in iPD [34,35]. However, the extent to which circadian rhythm disturbances affect the antioxidant activity in PD remains unclear. The ROS formation triggers mitochondrial dysfunction, followed by changes in the electron transport chain and increased damage to nuclear and mitochondrial DNA, thereby leading to neuroinflammation. These events result in a vicious cycle, with increased ROS formation and progressive neuron loss [36]. Mutations in circadian genes (*CLOCK, BMAL1, PER 1–3, CRY 1,* and *CRY 2*) are responsible for circadian rhythm dysregulation in PD, resulting in oxidative stress, neuroinflammation, metabolic dysfunction, and immunity suppression [5]. A few studies have suggested the association between the circadian rhythm and oxidative stress. For instance, the decrease in the *BMAL1* expression was associated with mitochondrial dysfunction and ROS overproduction [37]. Our results revealed that increasing ROS will decrease *SIRT1* and increase *BMAL1*, resulting in disruptions in the circadian rhythm and apoptosis of the SH-SY5Y cells. Therefore, apart from the traditional symptomatic management of neurodegenerative diseases, such as iPD, the alteration and modulation of the circadian rhythm might prove beneficial for modifying the disease process by increasing neuroprotection.

Epidemiological and dietary intervention studies have revealed neuroprotective effects in animal models from theaflavin’s antioxidant character, which is rich in black tea [13]. The citrus flavonoid hesperetin produced anti-inflammatory and antioxidative effects by regulating the Nrf2 and NF-kB expressions in a PD animal model [36]. For peripheral nerve injury, some extracts from Cannabis sativa L. were documented to restore muscle function by measuring the ratio between the gastrocnemius and tibialis anterior muscle [38]. Proanthocyanidin-rich foods, such as cranberries, cocoa, grapes, apples, strawberries, red wine, and green tea, may alleviate neurodegeneration in PD by enhancing mitochondrial activity [39]. 

Danshen, a common traditional Chinese medicine, has a demonstrated bioactivity in several organ systems and has been widely analyzed and applied in the treatment of several diseases. A study evaluating the effect of Danshen on the central nervous system reported that it exhibited neuroprotective effects and preserved cognitive function in Alzheimer’s disease and ischemic stroke animal models through different targets, resulting in decreased intracellular oxidative stress, apoptosis, inflammation, and platelet aggregation, apart from enhanced neurogenesis [40].

According to our results, DT exerted an increased cell viability on the SH-SY5Y cells, possibly due to reducing the oxidative stress, ROS formation, and apoptosis that subsequently rescued the circadian clock genes. The DT treatments reduced the 6-OHDA-induced ROS formation. Moreover, a significant increase in the cell viability and reduced apoptosis was observed in the DT-treated cells compared to the untreated cells. Our findings corroborated a study where Danshen also exhibited antioxidative effects by suppressing the ROS formation in a rotenone toxicity cellular model [41].

*SIRT1* is highly expressed in neurons and glial cells in the human brain [42], and the hippocampus and hypothalamus of the adult mouse brain [43,44]. *SIRT1* plays a role in neurodegenerative diseases [45] and is considered a suppressor of oxidative stress in PD [46]. Our study indicated that DT protected against neurotoxicity in a *SIRT1*-dependent manner. Nevertheless, further silencing and knockdown of *SIRT1* is still needed to confirm whether DT acts through the *SIRT1*–*BMAL* pathway. In this study, we demonstrated that *SIRT1* targeted and regulated the expression of the circadian clock genes *BMAL1*, *PER1,* and *CLOCK* in a PD model, thereby confirming the key role of *SIRT1* in PD pathogenesis. EX-527, an inhibitor of *SIRT1*, reversed the effect of DT, thereby indicating an association between DT and *SIRT1*. The apoptosis was enhanced after the *SIRT1* inactivation and *BMAL1* activation. The effect may be increased once *SIRT1* is activated.

In our study, NAC inhibited the production of ROS, and DT displayed similar results. At the same time, inhibiting ROS also concomitantly activated SIRT1 but inhibited BMAL1 and caspase-3. Inhibiting SIRT1 also reduced the utility of DT and reduced the cell viability.

As shown in Figure 6, silencing SIRT1 has the same result and enhanced the reduction in the cell viability. However, DT increased the SIRT1 activity and improved cell survival in the presence of 6-OHDA.

**Figure 7:**
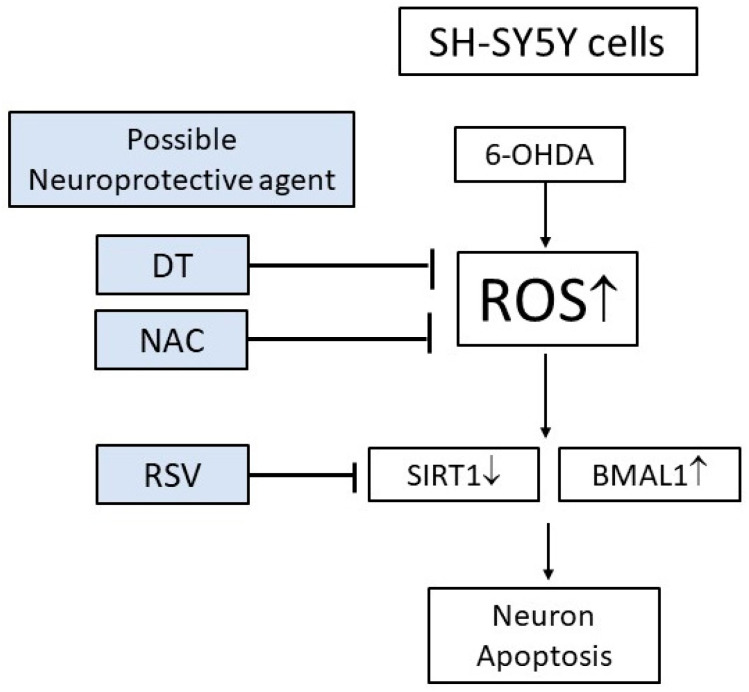
Schematic representation of the mechanisms of DT and the other agents reducing the SH-SY5Y cell death by inhibiting the ROS formation and enhancing the *SIRT1* expression.

We propose that ROS may be the key element for controlling the DT effects. DT and RSV displayed possible neuroprotective effects by activating SIRT1 and regulating the circadian clock genes, followed by reducing the death of the SH-SY5Y cells induced by 6-OHDA (Figure 7).

Our study had several strengths, including multiple adjustments and adequate experiments. Our study also had some limitations. First, no animal studies existed for more evidence in the circadian clock gene. Second, the SH-SY5Y cells were not purely dopaminergic since the cell line was obtained as a neuroblastoma derivative. The nature of this tumor resulted in physiological features that differed from those of normal dopaminergic neurons.

In conclusion, DT increased the expression of SIRT1 and attenuated the alteration of the circadian clock genes BMAL1 and PER1. DT exhibited a potent antioxidative activity as NAC, suggesting that it may act through the SIRT1–BMAL1 pathway [44]. ROS may be the key element for controlling the DT effects. DT and RSV activated SIRT1 and the circadian clock genes, and reduced the death of the SH-SY5Y cells induced by 6-OHDA. Therefore, DT regulated the molecular circadian clock genes, possibly through SIRT1. Thus, the in vitro studies of the active compound appeared promising. However, there is a need for further in vivo studies to establish the true efficacy and safety profile of the test compound before it can be applied in clinical practice.

## 4. Materials and Methods

### 4.1. Cell Culture and Treatment

The human neuroblastoma SH-SY5Y cells were obtained from the American Type Culture Collection (ATCC, Manassas, VA, USA) and grown using Dulbecco’s Modified Eagle Medium (DMEM) (ThermoFisher Scientific, Watham, MA, USA) supplemented with 10% fetal bovine serum, penicillin (100 U/mL), and streptomycin (100 U/mL) and incubated at 37 °C in an atmosphere of 5% CO_2_. The cells were seeded in 96-well plates at a density of 5.0 × 103 cells per well and incubated for 24 h. For the induction of the neurotoxicity, varying concentrations of 6-OHDA were added to each well, and the plates were incubated for 24 h. DT was added 30 min after 50 µM of the 6-OHDA treatment.

The SH-SY5Y cells were used for the transient knockdown of *SIRT1*. Briefly, the cells (4000 cells/well) were transfected with SiRNA Stealth siRNA or control siRNA (ThermoFisher Scientific, Watham, MA, USA) in each well by using 1 μL of Lipofectamine 2000 (Invitrogen, Camarillo, CA, USA) (ThermoFisher Scientific, Watham, MA, USA), according to the manufacturer’s instructions. Then, the cells were harvested after the transfection.

### 4.2. Real-Time Quantitative Reverse Transcription Polymerase Chain Reaction

A quantitative reverse transcription polymerase chain reaction (QRT-PCR) was applied extensively in genomics studies, especially where target-specific primers and real-time technology served a greater advantage [47]. By using the TRIzol reagent, the total RNA was isolated from the SH-SY5Y cells (Invitrogen, Camarillo, CA, USA) following the manufacturer’s instructions. The reverse transcription was performed using the cDNA Synthesis Kit (Roche Diagnostics GmbH, Mannheim, Germany). The cDNA sequence of the three circadian clock genes (*PER1*, *CLOCK*, *BMAL1*) and *SIRT1* were evaluated, and the specific forward and reverse primers and MGB TaqMan^®^ probe were designed using the Primer Express software version 1.5 (Applied Biosystems, Foster City, CA, USA). Polymerase chain reactions (PCRs) were performed using the 7900HT Fast Real-Time PCR System (Applied Biosystems, Foster City, CA, USA).

### 4.3. Evaluation of Apoptosis and the ROS Levels Using Flow Cytometry

The ROS is a molecule involved in oxidative reactions, immune systems, and the regulation of the cell cycle. The generation of ROS might indicate excessive oxidative damage inside the tested cell [48]. The SH-SY5Y cell line was seeded in a 100 mm plate and cultured overnight before treatment. After the 6-OHDA (30 or 50 μM) treatment, with or without DT 5 μM, the cells were detected for the cell cycle progression and necrosis/apoptosis using PI staining and an-nexin V/PI double staining, according to the manufacturer’s instructions. The stained samples were further analyzed using flow cytometry (BD Bioscience FacsCanto II flow cytometer, Marshall Scientific, Hampton, NH, USA).

The intracellular ROS levels were evaluated using 2′-7′dichlorofluorescin diacetate (DCFH-DA) as the fluorescent probe. In brief, the SH-SY5Y cells (1 × 10^5^ cells/well in six-well plates) were pretreated with 1 μM of DT for 2 h. The supernatant was removed, and the cells were incubated with 1 mL DCFH-DA (10 μM of DCFH-DA dissolved in serum-free DMEM) for 20 min at 37 °C in the dark. Subsequently, the cells were rinsed twice with phosphate-buffered saline (PBS) for detachment then resuspended in 1 mL PBS. The ROS levels were analyzed using flow cytometry (BD Bioscience FACSCanto II Flow Cytometer, Marshall Scientific, Hampton, NH, USA) and the values were expressed relative to the control, according to the manufacturer’s instructions.

### 4.4. Western Blotting

The protein analysis was established using Western blotting. The tested proteins were transferred and separated based on the molecular weight or discharge character [49]. The samples were prepared in a radioimmunoprecipitation assay buffer (20 mM Tris-HCl pH 7.5, 150 mM NaCl, 1 mM Na_2_EDTA, 1 mM EGTA, 1% NP-40, 1% sodium deoxycholate, 2.5 mM sodium pyrophosphate, 1 mM β-glycerophosphate, 1 mM Na_3_VO_4_, and 1 μg/mL leupeptin). For the Western blotting, 30 μg of total lysate was separated using 6–15% sodium dodecyl sulfate-polyacrylamide gel electrophoresis and transferred to the polyvinylidene difluoride membranes (Millipore, Darmstadt, Germany). The membranes were blocked with non-fat dry milk for 1 h and incubated overnight with 1:3000 diluted primary antibodies against the phosphorylated epitopes of caspase-3, *BMAL1*, *CLOCK*, and *SIRT1* (all purchased from Cell Signaling Technologies, Danvers, MA, USA). β-Actin (1:5000 dilution, Sigma-Aldrich, St. Louis, MO, USA) was used as an internal control. The secondary antibodies were horseradish peroxidase-conjugated goat anti-mouse IgG (Sigma-Aldrich) and goat anti-rabbit IgG (Sigma-Aldrich). The membranes were briefly incubated using Western Lightning Plus-ECL, an enhanced chemiluminescent substrate, (PerkinElmer Inc., Waltham, MA, USA) to visualize the proteins.

### 4.5. Experimental Set-Up

**Figure 8:**
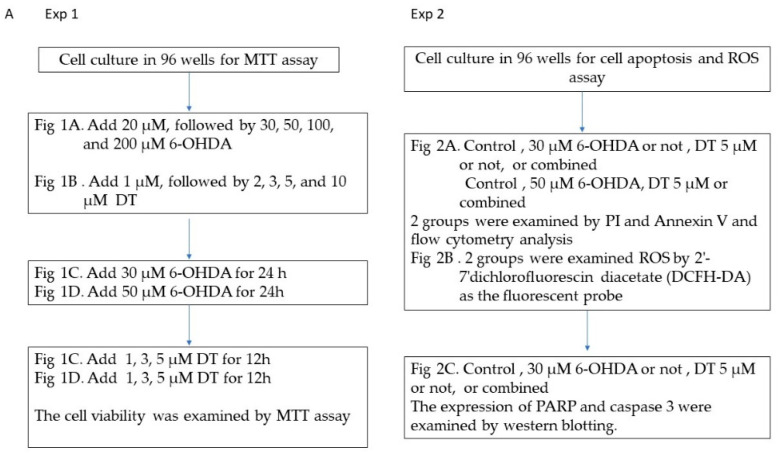

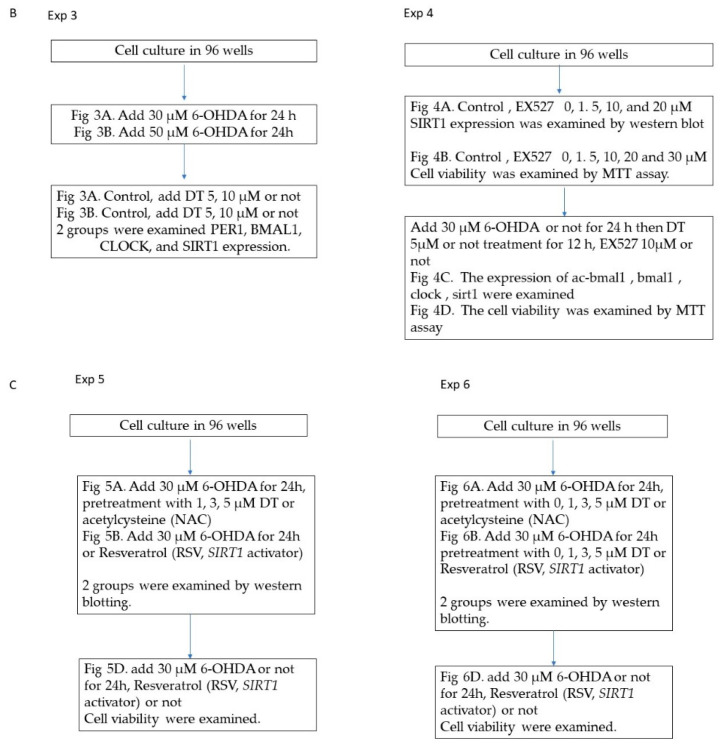
Flowcharts of experimental set-up.

The experimental procedure follows the flow chart below (Figure 8). 

### 4.6. Statistical Analyses

The data on the replicate samples (n = 3 to 6, depending on the experiment) were expressed as the mean ± the standard error of the mean, and the experiments were performed at least three times. The differences between the two groups were calculated using the unpaired two-tailed Student’s *t*-test, and a *p*-value < 0.05 was considered statistically significant. The statistical analyses were performed using SPSS 15.0 (SPSS, Chicago, IL, USA).

## Data Availability

All the data generated or analyzed during this study are included in this published article.
